# Investigation on Rupture Initiation and Propagation of Traffic Tunnel under Seismic Excitation Based on Acoustic Emission Technology

**DOI:** 10.3390/s22124553

**Published:** 2022-06-16

**Authors:** Xiling Liu, Yuan Zeng, Ling Fan, Shuquan Peng, Qinglin Liu

**Affiliations:** School of Resources and Safety Engineering, Central South University, Changsha 410083, China; lxlenglish@csu.edu.cn (X.L.); zydcsuyx@csu.edu.cn (Y.Z.); pqr97linger@csu.edu.cn (S.P.); 195512086@csu.edu.cn (Q.L.)

**Keywords:** traffic tunnel, shaking table test, acoustic emission, rupture initiation

## Abstract

Traffic tunnels are important engineering structures in transportation, and their stability is critical to traffic safety. In particular, when these tunnels are in an earthquake-prone area, the rupture process under seismic excitation needs to be studied in depth for safer tunnel design. In this paper, based on a construction project on the Nairobi-Malaba railway in East Africa, a laboratory shaking table test with 24 working cases of seismic excitation on a mountain tunnel is designed, and the acoustic emission (AE) technique is employed to investigate the tunnel rupture process. The results show that the high frequency components between 20 and 30 kHz of AE signals are the tunnel rupturing signals under the seismic excitation under such conditions. The tunnel vault and the arch foot are prone to rupture during the seismic excitation, and the initial rupture in the arch foot and vault of the tunnel occur under the horizontal and vertical Kobe wave seismic excitation, respectively, with a maximum acceleration of 0.4 g. After the rupture initiation, the tunnel arch foot continues to rupture in the subsequent working cases regardless of whether the excitation direction is horizontal or vertical, while the tunnel vault does not rupture continuously with the implementation of the subsequent excitations. Moreover, the Kobe seismic wave has a higher degree of damage potential to underground structures than the El seismic wave.

## 1. Introduction

Traffic tunnels are an important part of infrastructure in transportation around the world, and their stability is a vital factor affecting traffic safety. In particular, some traffic tunnels located in seismic active areas will be severely damaged by strong earthquakes. For example, the Daodao tunnel in Japan suffered large horizontal and vertical displacements during the earthquake [1]; 13 tunnels in Sichuan, China, were severely damaged during the 2008 Wenchuan earthquake [2]; and many longitudinal and vertical cracks were produced in the Melamchi tunnel during the Gorkha earthquake in 2015 [3]. Therefore, it is necessary to further study the rupture process under seismic excitation to better understand the response of tunnels to earthquakes, which is pivotal to the design of safe tunnels.

Due to site conditions and the unpredictability of earthquakes, the analysis of the seismic response of the tunnel can only be carried out after the earthquake, and the data collected on site can only be used to analyse the failure mode of the tunnel under the action of seismic waves. Meanwhile, seismic responses and rupture mechanisms of tunnels under the action of seismic waves are mostly studied through the shaking table system [4,5,6]. Usually, acceleration sensors and strain gauges are employed to monitor the acceleration and strain response characteristics of the tunnel in a shaking table test. By reviewing the monitoring data from the acceleration sensors and strain gauges, the mechanical and deformation response of the tunnel under the seismic excitation can be investigated [7,8,9,10]. In order to identify the damage of a tunnel in a shaking table test, some other monitoring techniques, such as white noise scanning, are also used to study crack propagation in the tunnel lining under the enhanced seismic excitation [11]. Regardless of the monitoring techniques employed in shaking table tests, it is recognized that the ruptures are always distributed in the vault, shoulder, wall, and bottom of the tunnel lining [2,9,12,13,14,15]. Also, the tunnel lining ruptures in vaults are usually distributed along the longitudinal and transverse direction of the tunnel [5,15,16,17]. Although various monitoring techniques are used in shaking table tests, they can hardly monitor the rupture initiation and propagation of the tunnel during the vibration excitation process. Even the white noise scanning technique can only identify the tunnel lining damage in a shaking table test, which has complex operation process and high cost. An in-depth investigation on rupture initiation and propagation of a tunnel under seismic wave excitation is needed, as it is crucial for understanding the seismic response mechanism and the tunnel seismic design. Therefore, a new monitoring technique should be employed in shaking table tests to analyse rupture initiation and propagation characteristics of tunnels under seismic excitation.

As an effective non-destructive testing method, acoustic emission (AE) technology has been widely used for real-time rupture monitoring. It can realize the real-time monitoring of the whole process of a structural life cycle rupture, and can also be used to evaluate the damage status of materials based on the extracted AE parameters. AE technology originated from earthquake research [18,19], and it is widely used in crack propagation, focal location, and quantitative damage research of indoor rock samples [20,21,22,23,24,25,26,27,28], as well as the stability monitoring of in situ rock mass [29,30,31,32]. Because of its good monitoring effect, AE technology is also used in the health monitoring of bridges, concrete structures, cementitious materials, steel structures, and many other engineering fields [20,33,34,35]. Thus, AE technology should also be able to monitor the tunnel rupture process in shaking table tests. However, since the complex experimental environment and the vibration process have a great influence on AE sensor coupling and signal collection, AE technology is rarely used for damage monitoring in shaking table tests. As the vibration signals of the shaking table will also be collected while the AE sensor collecting the rupture signals, the problem is how to distinguish between vibration signals and rupture signals, while the effective separation of the rupture signals from collected signals is the key to analyse tunnel rupturing under seismic excitation by AE technology. The vibration signal is similar to a low-frequency sinusoidal wave signal [33], while the rupturing signal is a burst-type pulse with a high-frequency component [19,33,36]. Therefore, a suitable signal analysis method needs to be employed to analyse the non-stationary AE signals in the shaking table test, which also requires further investigation.

In this research, we carried out a large-scale indoor tunnel shaking table test based on a construction project for the Nairobi-Malaba standard gauge railway (the Nairobi Malaba railway) in the transition zone from the Kapiti Plateau to the Great Rift Valley in Kenya. AE and deformation monitoring technology were used to investigate the seismic response characteristics of the tunnel. By distinguishing the vibration signals and the rupture signals of the tunnel collected by the AE sensors under the vibration load, rupture initiation under seismic excitation was determined. Then, the rupture propagation characteristics of the tunnel under different types of seismic waves were investigated according to the distinguished rupturing AE signals and the tunnel deformation data, and the rupture mechanism of the tunnel under seismic excitation was also deduced. The results in this paper will be of great help for an in-depth understanding of the tunnel rupture mechanism under seismic excitation.

## 2. Experimental Setup

### 2.1. Tunnel Similar Model Preparation

The prototype simulated in this paper is the tunnel of the Nairobi-Malaba standard gauge railway in Kenya, which passes through the Great Rift Valley in Africa. The dimensions of the tunnel prototype are listed in Table 1. The tunnel buried at a depth of 40 m crosses a 90-degree inclination fault with 10 m thickness.

The basic parameters of the tunnel model similarity ratios are determined by length, Young’s modulus, and acceleration, with values of 1/20,1/32.5, and 1.0, respectively, based on the maximum bearing weight of the shaking table and scales of tunnel prototype. The other similarity ratios have been deduced and are listed in Table 1. The frequency in the table refers to the main frequency. The El Centro wave and Kobe wave were chosen to simulate seismic waves.

**Figure 1:**
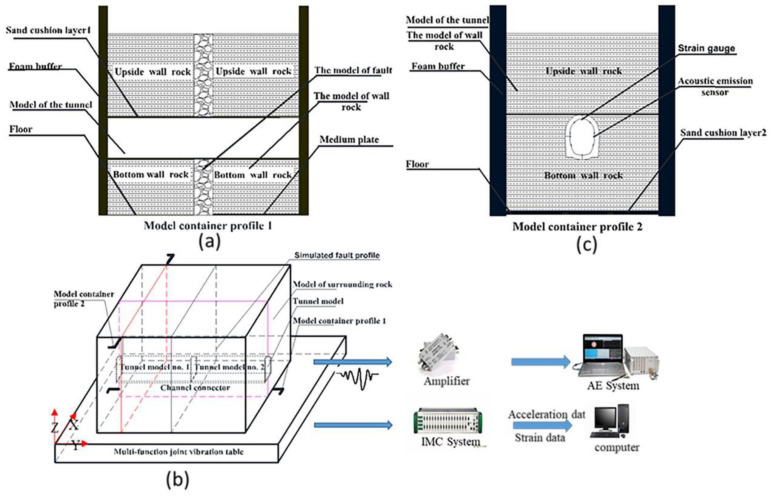
Schematic of experimental system: (**a**) side view of the model; (**b**) the overall design schematic of the experimental system; (**c**) the main view of the model.

A top-opening model box, tunnel lining with a fault, and surrounding rock were designed with a total weight of about 16 tons, as shown in Figure 1. The model box was 4 m long, 3.3 m wide, and 2.5 m high, welded by five 20 mm thick steel plates. According to the similarity ratios listed in Table 1, the tunnel segment with a lining thickness of 6 mm and a length of 1.2 m was made by mixing quartz sand, cement, barite powder, gypsum, and water with a weight ratio of 590:90:10:60:150. The surrounding rock was also made by the same materials, with a weight ratio of 590:35:10:60:150. The fault with 20 cm thickness and 90° inclination was perpendicular to the tunnel and filled with gravel, with particle sizes of less than 5 cm.

### 2.2. Deployment of AE Sensors and Strain Gauges

**Figure 2:**
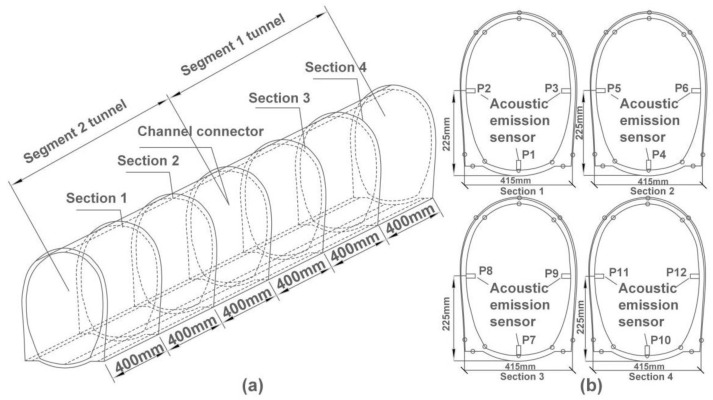
Deployment of AE sensors. The tunnel is divided into two parts (segment 1 tunnel and segment 2 tunnel), with channel connector as the boundary. (**a**) Locations of four tunnel sections for AE sensor deployment in tunnel inner lining; 6 AE sensors are mounted in each of segment 1 and segment 2 tunnel, and a total of 12 AE sensors are mounted on the surface of lining inner layer. (**b**) Locations of AE sensors on Section 1, Section 2, Section 3 and Section 4.

**Figure 3:**
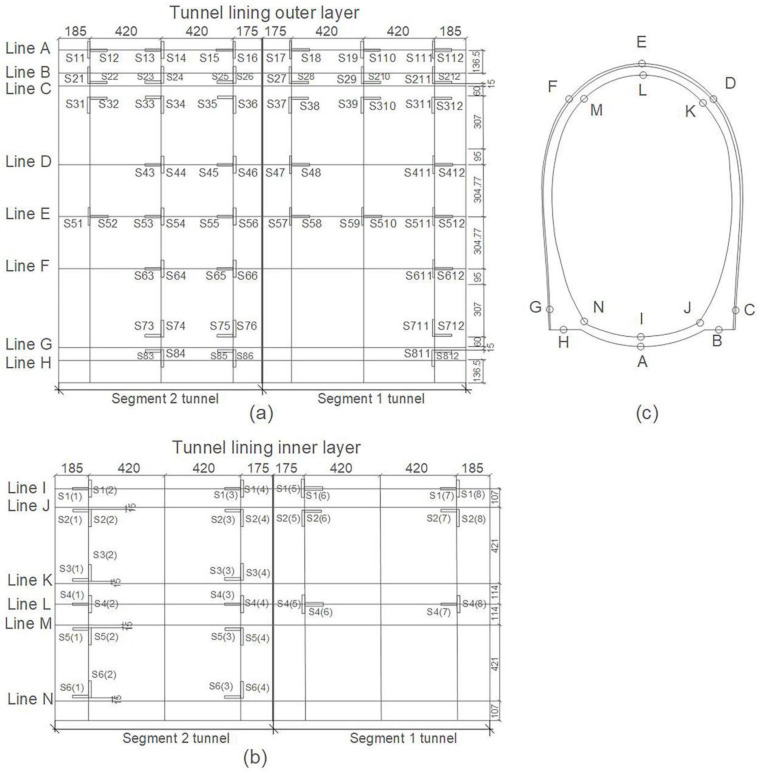
Layout of strain gauges. Combination marks of letter S and numbers are strain gauge numbers. A total of 112 strain gauges were deployed on the inner and outer tunnel lining. The data units in the figure are mm. (**a**) Tunnel outer lining; (**b**) tunnel inner lining; (**c**) tunnel cross section.

A PCI-2 AE instrument and 12 NANO-30 sensors with 140 kHz resonant frequency (produced by Physical Acoustic Emission Co. Ltd., Princeton Junction, NJ, USA) were used to monitor the fractures of the tunnel model excited by seismic waves. Twelve AE sensors (numbered as P1, P2, … P12) were uniformly arranged on four sections of the tunnel inner lining with a longitudinal spacing of 400 mm, as shown in Figure 2; the parameter setups of the AE instrument are listed in Table 2. A total of 112 strain gauges were deployed on the inner and outer tunnel lining, as shown in Figure 3.

### 2.3. Shaking Table System and Working Cases

A multi-functional shaking table test system for a high-speed railway of Central South University was used for seismic wave excitation. The shaking table system was 4 × 4 m in size and had a maximum bearing weight of 16 tons. It provided six degrees of freedom and three-way vibration, with a maximum displacement of 25 cm and a frequency of 0.5–40 Hz.

The El Centro wave and Kobe wave with main frequencies of 6.5 Hz and 6.2 Hz, respectively, were chosen to simulate seismic waves in the shaking table test. The El wave is the El Niño phenomenon recorded by El Centro Station during the 1940 California Empire Valley earthquake. The El wave has a duration of about 26 s and a frequency ranging from 2.0 Hz to 41.2 Hz, and the main frequency is 6.5 Hz. The El Centro wave is widely used in structural tests and classic seismic records for seismic response analysis. The Kobe wave was recorded by Kobe University Station in Japan in 2004. The Kobe wave has a duration of about 7 s and a frequency ranging from 1.1 Hz to 26.4 Hz, and the main frequency is 6.2 Hz. The direction of the input seismic wave in the shaking table test is along the X, Y, and Z axes; considering the scale and direction of seismic excitation, 24 working cases were designed, as shown in Table 3.

## 3. Results

### 3.1. Apparent Cracks

**Figure 4:**
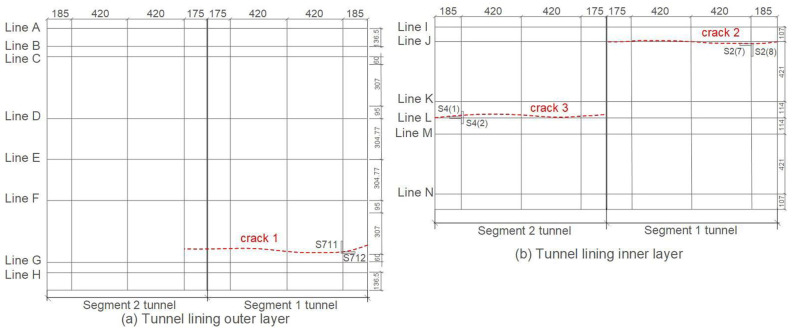
Schematic diagram of apparent cracks after test. Subpicture (**a**), Segment 1 tunnel: crack 1 appeared on the arch foot of the left sidewall of lining outer layer (Line G). Subpicture (**b**), Segment 1 tunnel: crack 2 appeared on the arch foot of the right sidewall of lining inner layer (Line J), and Segment 2 tunnel: crack 3 appeared on the vault of lining inner layer (Line L). The data units in the figure are mm.

After the shaking table test, three apparent cracks appeared on the arch foot at the non-connected end of the Segment 1 tunnel and on the vault of the Segment 2 tunnel. As shown in Figure 4, crack 1 appeared on the lining outer layer of Segment 1 tunnel, which is closer to strain gauges S711 and S712; crack 2 appeared on the lining inner layer of Segment 1 tunnel, which is closer to strain gauges S2 (7) and S2 (8); crack 3 appeared on the lining inner layer of Segment 2 tunnel, which is closer to strain gauges S4 (1) and S4 (2).

### 3.2. Variation of AE and Strain in Various Working Cases

There has been little data been collected by AE sensors from working cases 1–8 due to low vibration acceleration (with 0.2× *g* acceleration, as shown in Table 3), while each AE sensor collected a large amount of data starting from working case 9, which is where the specific analysis commenced. Furthermore, since the location of AE sensors P2, P3, P7, and P10 are near the ruptures (the layout of AE sensors can be found in Figure 2), the data collected by those four sensors have been used for rupturing analysis.

It is worth noting that, during the entire vibration test, AE sensors will collect the tunnel rupturing signals as well as the vibration signals of the shaking table; hence, the signals collected by AE sensors are the superposition of vibration and rupturing. The vibration signal is similar to a low-frequency sinusoidal wave signal, while the rupturing signal is a burst-type pulse with a high-frequency component. Therefore, it is only when the rupturing signal in waveform data (corresponding to a specific AE event) has been separated that the rupture initiation and propagation in the tunnel during vibration can be fully analysed. In the process of AE signal analysis, centroid frequency is the centre of mass of the power spectrum, which is also known as the first moment of inertia, and has always been used to evaluate the characteristics of the frequency component of a signal. When a collected AE signal has a significant high-frequency component, its centroid frequency will be higher, so the collected AE waveforms with high centroid frequency are more likely to contain a rupturing signal in a tunnel vibration test. Therefore, in this research, the waveform with the largest centroid frequency collected by the AE sensors P2, P3, P7, and P10 at each working case was screened out for rupture process analysis of tunnel under vibration.

**Figure 5:**
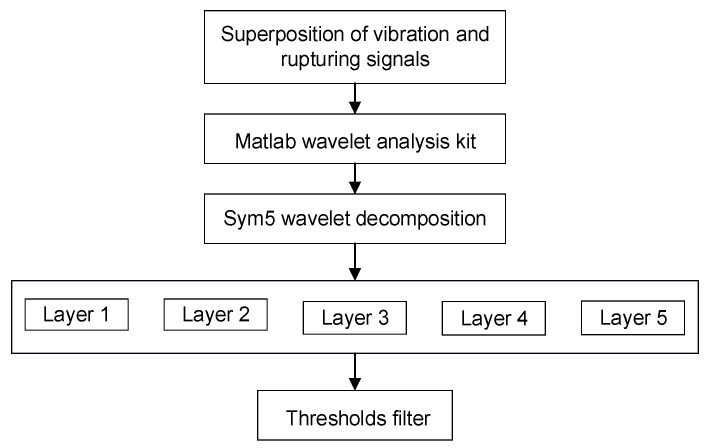
The flowchart of wavelet decomposition for vibration and rupturing superposition signals collected by AE sensors.

Furthermore, the AE signal is a kind of a non-stationary signal, especially in a shaking table test, where the signals collected by AE sensors are the superposition of vibration and rupturing. Therefore, a special technique should be employed for the analysis of such non-stationary AE signals. As a novel multi-resolution analysis method, wavelet transforms combine the frequency domain and time domain to appropriately represent the multi-scale characteristics of the signals [37], and they are particularly suitable for processing non-stationary signals. Then, wavelet de-noising and two-layer lifting wavelet decomposition and reconstruction techniques can be used to process the screened waveforms with the largest centroid frequency collected by the AE sensors P2, P3, P7, and P10. It was found that Daubechies and Symlets wavelet series are more suitable for AE signal analysis in dynamic loading tests [38], and Sym5 wavelet was considered to be the most suitable candidate for soft threshold filtering [39]. Thus, the signals collected by AE sensors was decomposed into five layers using the Sym5 wavelet, and the threshold processing was adjusted according to the noise level of wavelet decomposition in each layer. The specific wavelet decomposition flowchart is shown in Figure 5.

#### 3.2.1. Variation of AE and Strain at the Arch Foot

**Figure 6:**
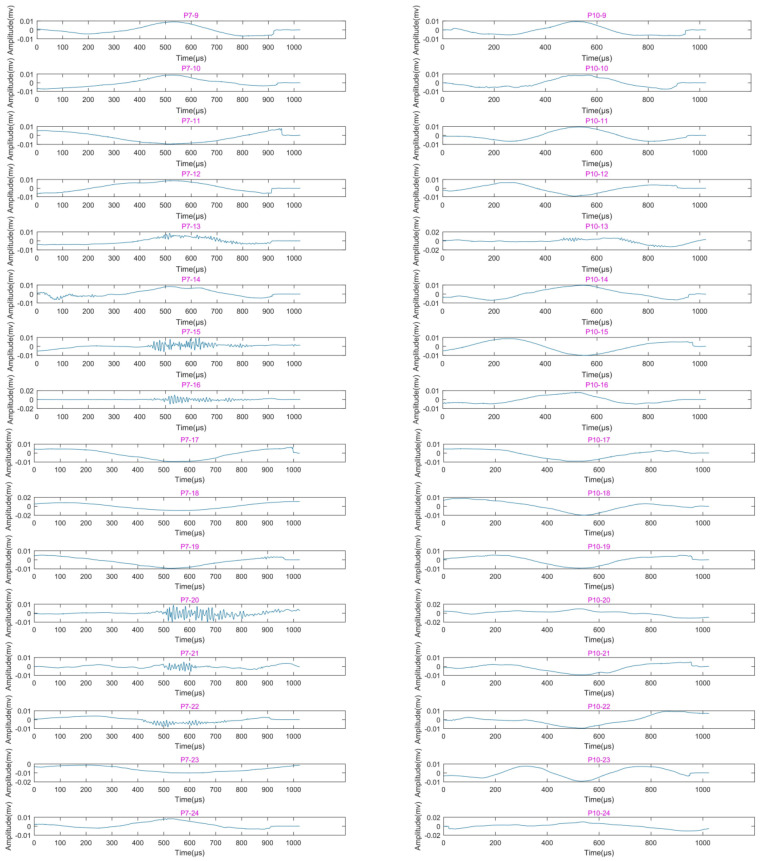
Reconstructed waveforms of AE signals with largest centroid frequency collected by sensors P7 and P10 in working cases 9–24 after wavelet decomposition and de-noising. The waveforms of sensors P7 and P10 for working case 13 (subpicture P7–13 and P10–13, respectively) contain high-frequency fluctuations, which indicates the initiation of ruptures in the tunnel arch foot.

**Figure 7:**
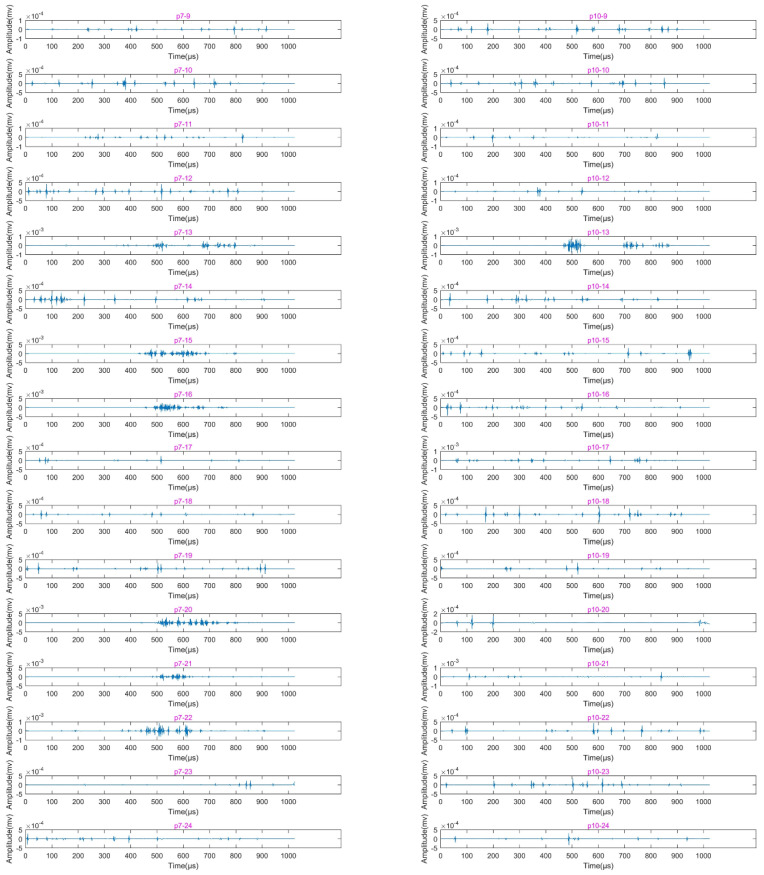
Reconstructed waveforms of signals with largest centroid frequency collected by sensors P7 and P10 in working cases 9–24 after lifting wavelet decomposition and filtering out the first layer low-frequency components. The waveforms of P7–13, P7–15, P7–16, P7–20, P7–21, P7–22, and P10–13 correspond to high-frequency fluctuations in various working cases shown in Figure 6.

Figure 6 shows the first-layer wavelet decomposition and reconstruction of the signals with largest centroid frequency collected by AE sensors P7 and P10 for working cases 9–24. It can be seen from Figure 6 that, for working cases 9–12 of sensors P7 and P10 (as shown in subpictures P7–9–P7–12 and P10–9–P10–12), only the shaking table vibration signals have been collected. In working case 13, both sensors P7 and P10 collected the signals with smooth vibration waveforms mixed with high-frequency fluctuations; the signals in cases 15, 16, 20, 21, and 22 of sensor P7 (as shown in subpicture P7–15, P7–16, P7–20, P7–21, and P7–22) also contain high-frequency fluctuations. Figure 7 shows the signals collected by sensors P7 and P10 for various cases after filtering out the low-frequency component. It is clear in Figure 7 that the amplitudes of signals in those cases with high-frequency fluctuations are all greater than 10^−3^ mv, which is an order of magnitude higher than the others. In addition, the strain value of the external strain gauge S711 on the left arch foot reached 134.29 in working case 13, as shown in Table 4 (a strain value in excess of 100 always indicates the occurrence of cracks). Based on the data from AE sensors and strain gauges, the ruptures in the arch foot may have started at working case 13; this will be further discussed in the following discussion section.

#### 3.2.2. Variation of AE and Strain at the Arch Vault

**Figure 8:**
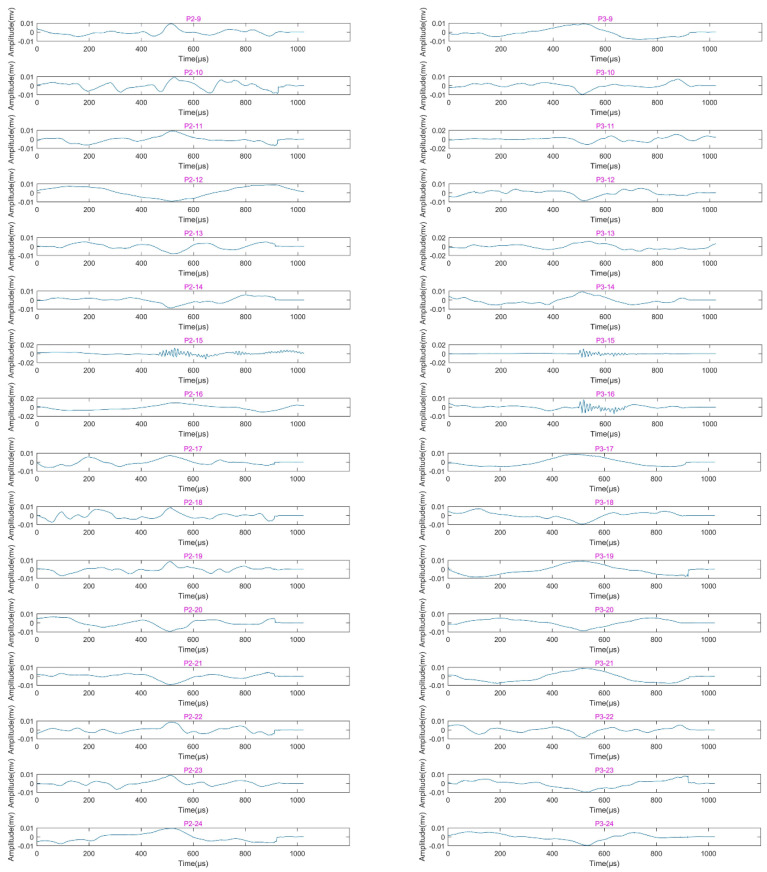
Reconstructed waveforms of AE signals with largest centroid frequency collected by sensors P2 and P3 in working cases 9–24 after wavelet decomposition and de-noising. The waveforms of sensors P2 and P3 in working case 15 (subpictures P2–15 and P3–15, respectively) contain high-frequency fluctuations, which indicates the initiation of ruptures in the tunnel arch vault.

**Figure 9:**
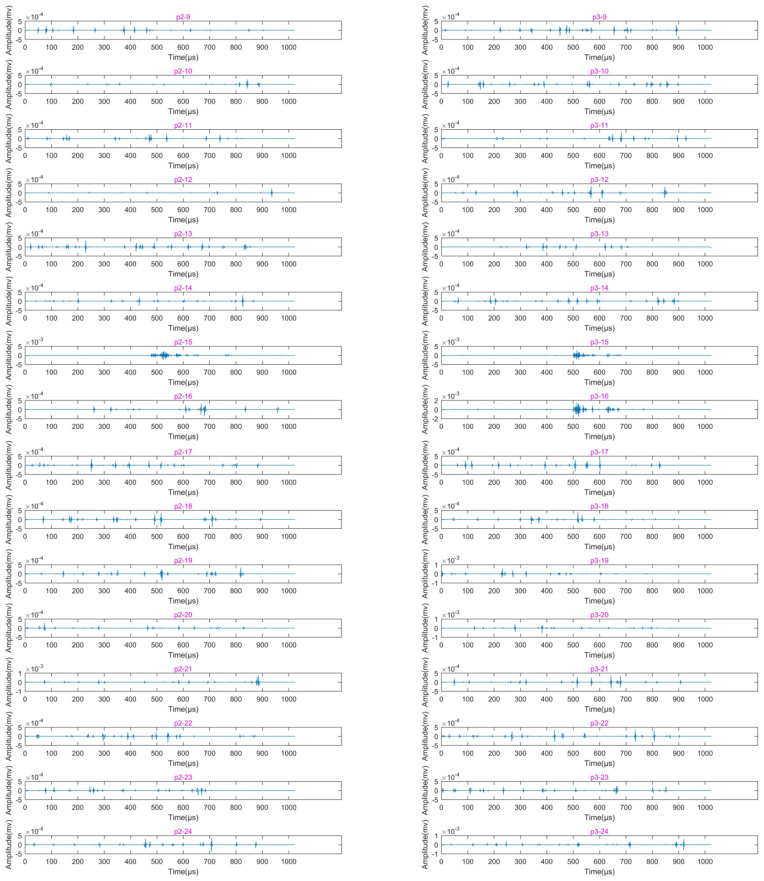
Reconstructed waveforms of signals with largest centroid frequency collected by sensors P2 and P3 in working cases 9–24 after lifting wavelet decomposition and filtering out the first-layer low-frequency components. The waveforms of P2–15, P3–15, and P3–16 correspond to high-frequency fluctuations in various working cases shown in Figure 8.

To obtain the rupture initiation cases of the arch vault of the Segment 2 tunnel, the lifting wavelet decomposition and reconstruction method were also used to process the AE signals. Figure 8 shows the first-layer wavelet decomposition and reconstruction of the signals with largest centroid frequency collected by AE sensors P2 and P3 for working cases 9–24. It can be seen from Figure 8 that only the shaking table vibration signals were collected by AE sensors P2 and P3 in working cases 9–14 (as shown in subpictures P2–9–P2–14 and P3–9–P3–14), while both sensors P2 and P3 collected the signals with high-frequency fluctuations in working case 15; the signals collected by P3 in working case 16 (as shown in subpicture P3–16) also contain high-frequency fluctuations. Figure 9 shows the signals collected by sensors P2 and P3 for various cases after filtering out the low-frequency components. It is clear in Figure 9 that the amplitudes of the signals in those cases with high-frequency fluctuations are all greater than 10^−3^ mv, which is an order of magnitude higher than the others. Furthermore, the strain value of the internal strain gauge S4(2) on the arch vault of the Segment 2 tunnel reached 158.7 in working case 15, as listed in Table 4, which indicates the occurrence of the ruptures. Based on the data from AE sensors and strain gauges, the ruptures in the arch vault may have started at working case 15; this will also be further discussed in the following discussion.

## 4. Discussion

### 4.1. Further Verification of the Tunnel Model Rupture Initiation

**Figure 10:**
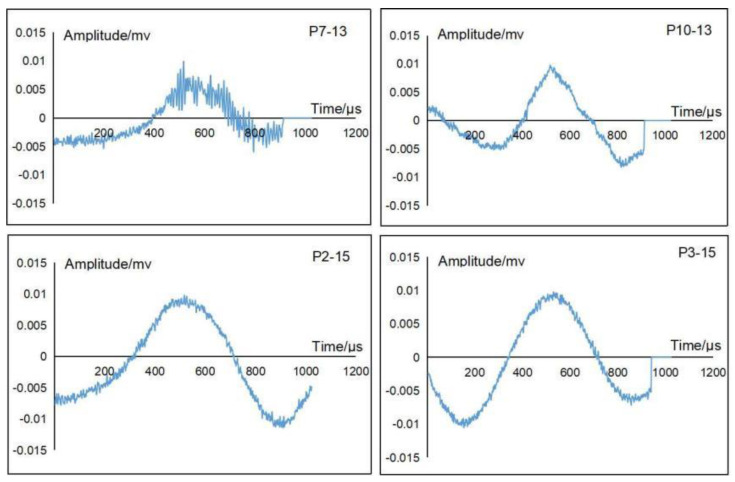
The original time domain AE waveforms of subpictures P7–13 and P10–13 in Figure 6, and those of subpictures P2–15 and P3–15 in Figure 8.

**Figure 11:**
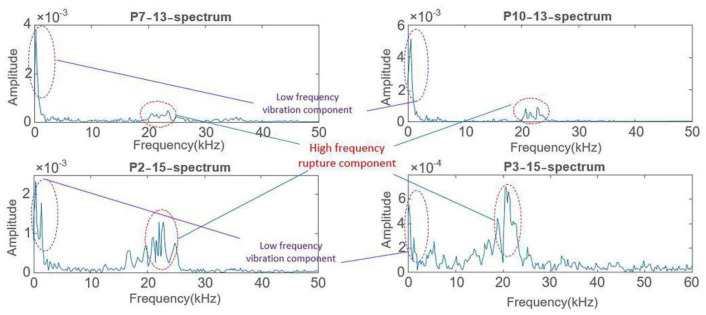
Spectra of the waveforms in subpictures P7–13 and P10–13 in Figure 6, and those in subpictures P2–15 and P3–15 in Figure 8. There are two peak intervals in each spectrum: the low-frequency peak interval is 1~3 kHz, whilst the high-frequency peak interval is 20~30 kHz.

According to the experimental results, it is known that the tunnel model may rupture on the arch foot and vault successively when the Kobe wave with 0.4× *g* gravitational acceleration is input in cases 13 and 15 in a horizontal and vertical direction, respectively. Therefore, we extracted the original AE waveforms of subpictures P7–13 and P10–13 in Figure 6, as well as subpictures P2–15 and P3–15 in Figure 8, as shown in Figure 10. It can be seen from Figure 10 that the signals near the arch foot (subpictures P7–13 and P10–13) and the signals near the vault (subpictures P2–15 and P3–15) are continuous-type signals. Usually, amplitude and count extracted from time domain AE waveform are two important parameters often used for AE signal analysis [40]. The amplitudes and counts of AE waveforms for P7–13, P10–13, P2–15, and P3–15 in Figure 10 are 40 dB and 1, 40 dB and 1, 41 dB and 1, and 40 dB and 1, respectively. Obviously, there is not much differences in amplitudes, nor the counts. We need other parameters to analyse the tunnel rupture under seismic excitation. As a matter of fact, the inferred rupture initiation in Section 3 is based on the frequency characteristics of the collected signals; therefore, it is necessary to perform spectrum analysis for further verification. Figure 11 shows the spectra of the waveforms in subpictures P7–13, P10–13, P2–15, and P3–15 in Figure 10. It is clear that each spectrum contains two peak intervals: the low-frequency peak interval and the high-frequency peak interval. The low-frequency peak interval is obviously the vibration component, while the high-frequency peak interval is between 20 and 30 kHz which is exactly within the frequency range of the laboratory concrete materials-rupturing AE signals [33,41].

Another way to verify the rupture initiation is to perform signal location. As shown in Figure 4, the apparent crack 3 in the arch vault of the Segment 2 tunnel can be located by sensors P2 and P3. The tested wave velocity in the concrete tunnel model was about 3000 m/s. The time difference of the signals collected by sensors P2 and P3 in working case 15 (the signals in subpictures P2–15 and P3–15 in Figure 8) is 3.43 μs, which corresponds to about 1 centimetre difference from the rupture source to sensors P2 and P3, and is consistent with the location of apparent crack 3 in Figure 4. This confirms that the AE signals are generated by rupturing in the arch vault. However, since the crack inside the arch foot did not appear in the line between P7 and P10, it is not feasible to determine the crack location generated at the arch foot. So far, we can conclude that the rupture in the arch foot initiated at working case 13, and the rupture in the arch vault initiated at working case 15.

### 4.2. Failure Mechanism of Tunnel during Earthquake

As shown in Table 4 and Figure 3, the strain gauges with relatively larger strain values are all distributed in the vault and arch foot, which indicates that the tunnel model is prone to failure at these two positions. This result is similar to the damage of a large tunnel model with or without voids on the top of the lining in a shaking table test performed by Xin et al. [17]. This is also verified by actual tunnel seismic damage; for example, the Longxi tunnel showed evidence of serious longitudinal and transverse rupturing and peeling at the vault and inverted arch after the Wenchuan earthquake [42]. In the shaking table test of this research, the monitoring data show that the horizontal excitation is very effective for increasing the seismic strain response of the abutment and vault. It can be seen in Figure 6 and Figure 7 that, after the damage to the arch foot in working case 13, the AE sensors still collected the rupture signals which could be extracted out of the maximum centroid frequency events of working cases 15 and 16 (Kobe wave with 0.4× *g* and X direction input) and working cases 20–22. This indicates that, after the initial rupture of the tunnel model at the arch foot in working case 13, the arch foot continues to rupture in the subsequent working cases, regardless of whether the vibration direction is horizontal or vertical. While the rupture of the vault only occurred in the working cases 15 and 16, the signals of the maximum centroid frequency events of each working case collected by the AE sensors did not include the rupture signals after working case 16 (as clearly seen in Figure 8 and Figure 9). From the different vibration response characteristics of the arch foot and the vault in the continuous shaking table test, it was shown that the tunnel vault and the arch foot are prone to rupture during vibration. However, the damage of the arch foot will continue to deteriorate with the subsequent vibrations after the initiation of rupture. Therefore, great attention should be paid to the arch foot for an earthquake-resistant tunnel design.

Furthermore, in the experiments of this research, Kobe wave and El wave were used in various working cases, but when the two types of seismic waves are input with the same magnitude, acceleration, and direction to drive the vibration of the shaking table, the Kobe wave caused the shaking table to make the tunnel model generate more ruptures (as shown in Figure 6 and Figure 8; the ruptures that occurred in working cases 13, 15, 16, 21, and 22 were all excited by the Kobe wave, while only ruptures that occurred in working case 20 were excited by the El wave). The Kobe wave has higher energy and shorter duration compared with the El wave, while El wave has rich high-frequency components [43,44,45,46]. Therefore, a seismic wave with high energy, short duration, and significant low-frequency components has a higher degree of damage potential to underground tunnels.

**Figure 12:**
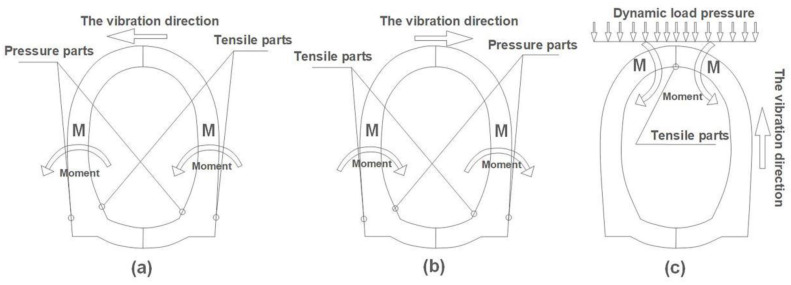
The schematic force diagram of tunnel model subjected to dynamic load in horizontal and vertical directions. (**a**,**b**) Vibration load in horizontal direction; compressive stress and tensile stress parts appear inside and outside the arch foot. (**c**) Vibration load in vertical direction; tensile stress part appears inside the vault.

Hashash et al. [47] suggested that the structures buried in soil or rock are bound by the underground medium, their deformation is mainly determined by the displacement field of the surrounding medium, and the physical and mechanical properties of the surrounding medium have significant effects on the seismic response of an underground structure. Therefore, when the tunnel model is horizontally vibrated, the tunnel shoulder and tunnel wall can be regarded as a cantilever beam model with a force under the action of the moment, and the maximum moment of the cantilever beam generates at the fixed end (arch foot) with the load. Moreover, the outside parts of the left arch foot and the inside parts of the right arch foot, or the outside parts of the right arch foot and the inside parts of the left arch foot, suffer two stress states of tension and compression which frequently change between each other during the vibration process (as shown in the schematic force diagrams in Figure 12a,b). Thus, the tensile stress zone in the arch foot of the tunnel model will be prone to damage during horizontal vibration. The experimental results show that, when a Kobe wave of 0.4× *g* is input from a horizontal direction (*X* axis) as in working case 13, the outside of the arch foot (Line G in Figure 4) and the inside of the arch foot (Line J in Figure 4) where the ruptures occurred are exactly the tensile stress zone, which correspond well with the theoretical analysis.

Also, when a Kobe wave of 0.4× *g* is input from a vertical direction (*Z* axis) as in working case 15, the tunnel model is subjected to a vertical dynamic load, and the vault can then be approximately regraded as a model of a simply supported beam (as shown in Figure 12c). A simply supported beam is subjected to the maximum moment in the middle of the beam and the stress-bearing side of the beam is subjected to compressive stress, while the bending side is subjected to tensile stress. Therefore, the seismic wave excitation in vertical direction makes the lining inner layer of the tunnel vault easier to break; in fact, the ruptures did occur on the lining inner layer of the tunnel vault (Line L in Figure 4) in working case 15 in this shaking table test.

## 5. Conclusions

(1) As an effective non-destructive testing method, AE technology is also suitable for tunnel rupture process monitoring in shaking table tests, and also rupture initiation and propagation characteristics of the tunnel, which are vital for deep understanding of dynamic responses of tunnels under seismic wave excitation. For the analysis of AE signals, centroid frequency can well characterize the distribution state of AE signal frequency components; it is an effective parameter to pick out waveforms which contain rupture AE signals in shaking table test. Furthermore, wavelet de-noising and two-layer lifting wavelet decomposition and reconstruction techniques are particularly suitable for non-stationary AE signals processing in complex shaking table tests, where the signals collected by AE sensors are the superposition of vibration and rupturing. Through wavelet decomposition and reconstruction, the rupture signals of the tunnel can be well separated from the vibration signals; the rupture AE signal frequency of the tunnel under seismic excitation in this research was found to be in the range of 20–30 kHz. However, the AE signal frequency is significantly related to the distance from rupture source to sensor, material properties, and the sensor resonant frequency; therefore, caution should be aroused when frequency characteristics of the tunnel model-rupturing AE signal in this research is used to make comparative analysis by other researchers.
(2) The vault and arch foot of the tunnel model are prone to rupture under seismic excitation in shaking table test. However, the damage of the arch foot will continue to deteriorate with the subsequent vibrations after the initiation of rupture, while the ruptures in vault do not continue to expand under the subsequent seismic excitation after the initiation of rupture. This indicates that arch foot and vault of the tunnel have different dynamic responses to seismic excitations. In addition, the results in this research show that the Kobe wave drive the shaking table to make the tunnel model generate more ruptures than the El wave, which means that the seismic wave with high energy, short duration, and significant low-frequency components has a higher degree of damage potential to underground tunnels. Therefore, great attention should be paid to the arch foot for underground tunnel design in earthquake-prone areas; also, materials with good resistance to seismic wave with short duration and significant low-frequency components should be selected as much as possible for tunnel construction in earthquake-prone areas.

## Figures and Tables

**Table 1:** Similarity ratios of the tunnel and surrounding rock.

Parameter		Prototype	Similarity Ratio	Model	Parameter	Prototype	Similarity Ratio	Model
Geometry (m)		48	$C_{l}$	2.4	Buried depth (m)	40.00	$C_{l}$	2.00
Active fault thickness (m)		7.0	$C_{l}$	0.35	Section height (m)	9.59	$C_{l}$	0.48
Young’s modulus/GPa		6.50	$C_{E}$	0.20	Sectional area (m^2^)	60.99	${C_{l}}^{2}$	0.15
Density (kg/m^3^)		2.4	$C_{E}/C_{l}C_{A}$	1.92	Lining thickness (m)	0.4	$C_{l}C_{E}^{-1/3}$	0.062
Time (s)		30	$\sqrt{C_{l}/C_{A}}$	7.19	Acceleration (g)	0.20	$C_{A}$	0.20
Frequency (Hz)	El wave	6.5	$1/\sqrt{C_{l}/C_{A}}$	29.07	Velocity (m/s)	58.8	$C_{A}\sqrt{C_{l}/C_{A}}$	13.15
	Kobe wave	6.2	$1/\sqrt{C_{l}/C_{A}}$	27.73				

$C_{l}$, $C_{E}$, and $C_{A}$ are 1/20, 1/32.5, and 1.0 respectively.

**Table 2:** Acquisition parameter settings of AE system.

Threshold (dB)	Analogue Filter (kHz)	Sample Rate (kHz)	Pre-Trigger (μs)	PDT (μs)	HDT (μs)	HLT (μs)
40	1~1000	2000	256	50	200	300

**Table 3:** Working cases of the shaking table test.

Working Cases	Seismic Wave	Scale	X Direction	Y Direction	Z Direction
1	El wave	0.2 × g	1	/	/
2		0.2 × g	/	1	/
3		0.2 × g	/	/	1
4		0.2 × g	1	1	/
5	Kobe wave	0.2 × g	1	/	/
6		0.2 × g	/	1	/
7		0.2 × g	/	/	1
8		0.2 × g	1	1	/
9	El wave	0.4 × g	1	/	/
10		0.4 × g	/	1	/
11		0.4 × g	/	/	1
12		0.4 × g	1	1	/
13	Kobe wave	0.4 × g	1	/	/
14		0.4 × g	/	1	/
15		0.4 × g	/	/	1
16		0.4 × g	1	1	/
17	El wave	0.6 × g	1	/	/
18		0.6 × g	/	1	/
19		0.6 × g	/	/	1
20		0.6 × g	1	1	/
21	Kobe wave	0.6 × g	1	/	/
22		0.6 × g	/	1	/
23		0.6 × g	/	/	1
24		0.6 × g	1	1	/

**Table 4:** Increment strains at vault and foot.

Working Cases	S4(2)	S711	S3(4)	S5(4)	S64	S44
1	10.987	47.61	13.43	25.64	34.18	39.06
2	8.545	57.38	29.29	56.16	67.14	35.41
3	9.766	41.51	34.18	37.85	54.93	41.51
4	8.55	48.83	69.58	61.04	69.10	54.57
5	9.766	57.371	14.65	34.18	89.11	42.73
6	0.00	10.987	35.40	56.16	73.31	48.83
7	0.00	30.519	26.86	45.17	90.34	37.84
8	2.44	10.99	39.06	67.14	68.61	48.83
9	9.77	52.49	20.75	18.31	24.42	13.43
10	15.87	19.53	85.45	48.83	70.81	24.42
11	20.75	76.91	47.61	36.62	28.08	23.19
12	13.43	32.961	92.78	62.26	73.25	31.74
13	18.31	134.29	21.97	29.30	48.83	30.52
14	24.42	173.35	87.90	84.23	70.81	53.71
15	158.7	148.93	52.49	51.27	47.61	29.30
16	126.96	393.09	96.44	95.22	85.74	51.27
17	15.87	125.74	31.74	37.84	35.40	21.97
18	37.84	275.9	103.77	84.23	93.99	29.30
19	283.22	198.99	74.47	92.78	54.93	24.42
20	175.79	277.12	111.09	113.53	91.56	31.74
21	202.65	365.01	45.17	51.27	67.14	52.49
22	274.67	355.25	106.21	44.05	85.45	61.04
23	1567.48	449.25	80.57	126.96	78.13	40.29
24	636.03	976.62	91.56	39.16	45.28	50.05

S4(2) and S711 are the strain gauges through which crack 3 and crack 1 passed, as shown in Figure 4. S3(4), S5(4), S64, and S44 are the selected strain gauges with relatively larger strain values. The specific locations of all strain gauges can be found in Figure 3.

## Data Availability

Data are contained within the article.
